# Characterisation of E12/E47 expression in colorectal cancer

**DOI:** 10.1186/1753-6561-9-S1-A45

**Published:** 2015-01-14

**Authors:** AS Chugha, L Flanagan, F Bane, L Young, B D’Orsi, JHM Prehn

**Affiliations:** 1 Department of Physiology and Medical Science, Centre for the Study of Neurological Disorders, Royal College of Surgeons in Ireland; 2 Endocrine Oncology Research Group, Department of Surgery and Epidemiology, Royal College of Surgeons in Ireland

## Background

In the Western world colorectal cancer is one of the leading causes of cancer-related mortality, and the second most common cancer in women and the third in men. Identification of prognostic markers capable of predicting patient response to chemotherapy is essential to determine more personalised and efficient treatment strategies in colorectal cancer, and may help identify novel therapeutic targets. The aim of the study was to characterise the expression of the E2A transcriptional regulator proteins, E12/E47 (also known as TCF-3 or E2A) in colorectal cancer patient tissue samples. Primarily E12/E47 antibody optimisation was required to allow us to subsequently examine E12/E47 protein expression in colorectal cancer tissue and investigate any correlation with patient outcome.

## Methods

Candidate antibodies directed against the E12/E47 protein were first evaluated by western blotting and densitometry assays on four different types of human colon cancer cell lines. Surgically resected colorectal tumours were obtained from 198 patients presenting with Dukes B and C colorectal cancer. Tissue microarrays were constructed from Formalin-Fixed Paraffin-Embedded samples. Tissue microarrays were then stained with varying dilutions of the candidate antibodies to establish a reliable and reproducible staining protocol.

## Results

In order to optimise the E12/E47 staining on tissue samples, two different antibodies, a rabbit monoclonal TCF-3 antibody (Cell Signaling) and a rabbit polyclonal E2A antibody (Santa Cruz) were used. Serial dilutions of these antibodies were made to determine the optimal concentration of antibody to use. Using immunohistochemistry, the first antibody did not detect any specific signal while a nuclear/cytoplasmic localisation of E12/E47 was identified in some tissue samples using the E2A antibody. This staining was scored in line with the Allred Immunohistochemistry Scoring system.

## Conclusions

Via antibody and protocol optimisation we were able to establish a protocol to specifically identify E12/E47 protein in human colorectal cancer tissue microarrays. This enables us to identify whether a correlation between E12/E47 expression in colorectal cancer patient tissue exists and whether it expression is linked to tumour aggressiveness. The identification of novel colorectal cancer markers may be relevant for diagnosis and treatment of cancer patients.

